# VB-MK-LMF: fusion of drugs, targets and interactions using variational Bayesian multiple kernel logistic matrix factorization

**DOI:** 10.1186/s12859-017-1845-z

**Published:** 2017-10-04

**Authors:** Bence Bolgár, Péter Antal

**Affiliations:** Department of Measurement and Information Systems, Budapest University of Technology and Economics, Magyar tudósok krt. 2., Budapest, 1117 Hungary

**Keywords:** Drug-target interaction prediction, Matrix factorization, Multiple kernel learning, Variational Bayes, Probabilistic graphical models

## Abstract

**Background:**

Computational fusion approaches to drug-target interaction (DTI) prediction, capable of utilizing multiple sources of background knowledge, were reported to achieve superior predictive performance in multiple studies. Other studies showed that specificities of the DTI task, such as weighting the observations and focusing the side information are also vital for reaching top performance.

**Method:**

We present Variational Bayesian Multiple Kernel Logistic Matrix Factorization (VB-MK-LMF), which unifies the advantages of (1) multiple kernel learning, (2) weighted observations, (3) graph Laplacian regularization, and (4) explicit modeling of probabilities of binary drug-target interactions.

**Results:**

VB-MK-LMF achieves significantly better predictive performance in standard benchmarks compared to state-of-the-art methods, which can be traced back to multiple factors. The systematic evaluation of the effect of multiple kernels confirm their benefits, but also highlights the limitations of linear kernel combinations, already recognized in other fields. The analysis of the effect of prior kernels using varying sample sizes sheds light on the balance of data and knowledge in DTI tasks and on the rate at which the effect of priors vanishes. This also shows the existence of “small sample size” regions where using side information offers significant gains. Alongside favorable predictive performance, a notable property of MF methods is that they provide a unified space for drugs and targets using latent representations. Compared to earlier studies, the dimensionality of this space proved to be surprisingly low, which makes the latent representations constructed by VB-ML-LMF especially well-suited for visual analytics. The probabilistic nature of the predictions allows the calculation of the expected values of hits in functionally relevant sets, which we demonstrate by predicting drug promiscuity. The variational Bayesian approximation is also implemented for general purpose graphics processing units yielding significantly improved computational time.

**Conclusion:**

In standard benchmarks, VB-MK-LMF shows significantly improved predictive performance in a wide range of settings. Beyond these benchmarks, another contribution of our work is highlighting and providing estimates for further pharmaceutically relevant quantities, such as promiscuity, druggability and total number of interactions.

**Electronic supplementary material:**

The online version of this article (doi:10.1186/s12859-017-1845-z) contains supplementary material, which is available to authorized users.

## Background

Drug-target interactions (DTI) or compound-protein interactions (CPIs) have become a focal point in chemo- and bioinformatics. There are many factors behind this trend, such as the direct, quantitative nature of bioactivity data [1], its unprecedented amount, public availability [2, 3], and variety including also phenotypic and content-rich assays and screenings [4]. Further factors are the semantic, linked open nature of the data [5, 6], collaborative initiatives in the pharmaceutical policy [1] and the construction of DTI benchmarks [7–13].

An additional factor is the varying granularity and multiple facets of the DTI task: it was already attacked in the 90’s in single target scenarios, e.g. by using neural networks of that time [14] and subsequently by kernel methods [15, 16]. A series of similarity-based methods were also developed for virtual screening [17–19]; in the early 2000’s molecular docking became popular [20, 21]; from the late 2000’s matrix factorization methods were developed [7, 22, 23]. As the importance of data and knowledge integration in drug discovery was further emphasized [1, 24–26], the incorporation of prior knowledge in DTI became mainstream and indeed improved predictive performance [23, 27–29].

Computational data and knowledge fusion approaches in the DTI problem seem to be especially relevant, as the growth of DTI datasets is limited by experimental and publication time and cost, while the cross-linked repertoire of side information expands at an enormous rate. This grand pool of information complementing the DTI data and the full scope of the DTI fusion challenge is best illustrated by the drug repositioning problem [30, 31]. In repositioning, i.e. in the finding of a novel indication for an already marketed drug, extra information sources could also be used, such as off-label drug usage patterns, patient-reported adverse-effects and official side-effects [32]. Notably, this information pool can be linked back to early stage compound discovery [33].

In this paper we investigate the multiple kernel-based fusion approach to the DTI task from a computational fusion perspective, by adopting widely used benchmark datasets, implementations and evaluation methodologies from Yamanishi et al. [7], Gönen [22], Pahikkala et al. [8] and Liu et al. [34]. Our contributions are as follows:

1. *VB-MK-LMF:* We present a Bayesian matrix factorization method with a novel variational Bayesian approximation, which unifies multiple kernel learning, importance weight for (positive) observations, network-based regularization and explicit modeling of probabilities of drug-target interactions.
2. *Effect of multiple kernels:* We report the results of a comparison against three leading solutions using two benchmark datasets, in which VB-MK-LMF achieved significantly better performance in most settings. We systematically investigate factors behind its performance, such as the type of the kernels, the role of neighborhood restriction and Bayesian averaging. Finally, we evaluate the effect of priors using varying sample sizes highlighting the regions where using side-information improves predictive performance.
3. *Posteriors for promiscuity and druggability:* We show that probabilistic predictions from VB-MK-LMF can be used to quantify the expected values for promiscuity or the number of hits in a DTI task.
4. *Dimensionality of the unified “pharmacological” space:* We investigate the learned unified latent representations of drugs and targets, and contrary to many studies we argue that drastically smaller dimensions are sufficient. We discuss the possibility that this low dimension, around 10, could be utilized in visual analytics and exploratory data analysis.
5. *Accessibility:* We report the adaptation of the developed variational Bayesian approximation to general purpose graphics processing units (GP-GPU). Evaluations show that 30× speed-up can be achieved using a standard GP-GPU environment. To support the development of current DTI benchmarks towards “computational DTI fusion”, we release the applied kernels, code and parameter settings for academic use.

**Fig. 1:**
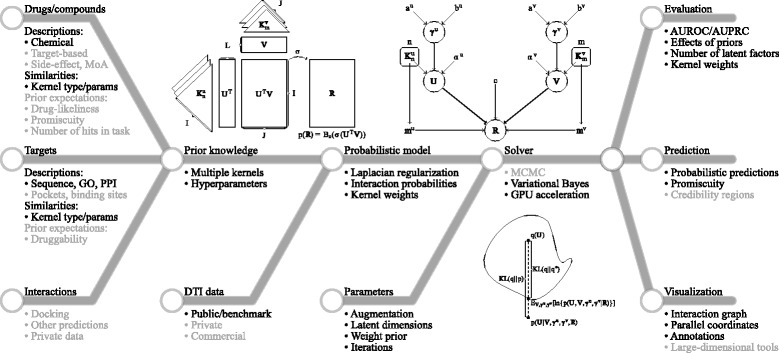
Overview of the VB-MK-LMF workflow. A priori information (left) are combined with DTI data through a Bayesian model (middle). Learning is carried out using a Variational Bayesian method which approximates the latent factors and optimal kernel weights. The model provides quantitative predictions of interaction probabilities and estimates of drug promiscuity (right). Finally, VB-MK-LMF supports the visualization and exploration of the unified “pharmacological” space. Gray indicates functionalities which may also be utilized in the VB-MK-LMF model but not explored in this paper

Figure 1 shows the overview of Variational Bayesian Multiple Kernel Logistic Matrix Factorization (VB-MK-LMF).

### Related works

To give an overview about related, earlier works [7, 27–29, 35–54], we summarize the main properties of their applied datasets, side information, methods and evaluation methodologies in Additional file 1).

#### DTI data

Drug-target interaction data has become a fundamental resource in pharmaceutical research, which can be attributed to its public availability in an open linked format, see e.g. [1, 5, 6, 55–58]. The relative objectivity of interaction activities and the side information about drugs and targets renders a unique status to the comprehensive tabular DTI data, even compared to media and e-commerce data [59], despite the issues of quality [60, 61], duality of commercial and public repositories [62–64] and selection bias related to the lack of negative samples [12] and promiscuity [65]. However, at present the heterogeneous, real-valued activity data are usually treated as binary relations, even though the use of raw data together with information about the measurement context is expected in more realistic DTI prediction scenarios [8, 46, 52]. Another largely overlooked property of the binary drug-target interaction data is its possibly indirect nature, which influences the applicable target-target similarities, e.g. in the indirect case protein-protein networks may have relevance (for the explicit treatment of direct and indirect relations, see e.g. RBM [45]).

#### DTI prior knowledge

The molecular similarity property principle [66, 67], the drug-likeliness of a compound [68, 69] and druggability of proteins [70] are essential concepts in the broader drug discovery context, together with molecular docking [20, 21] and binding site, pocket predictors [71], if structure information is available. However, their use as priors in the computational DTI task is still largely unexplored. If the goal is the discovery of indirect drug-target interactions, possibly including multiple paths, which are especially relevant in polypharmacology [72], then the use of molecular interaction and regulatory networks alongside protein-protein similarities is another open issue.

Chemical similarity, the most widespread source of prior knowledge in DTI, was the basis of many “guilt-by-association” approaches in chemo- and bioinformatics. Earlier investigations helped to understand the use of multiple, heterogeneous representations, similarity measures and introduced the concept of fusion methods in ligand-based virtual screening [17, 18, 73–75]. Beyond chemical similarities, target-based similarities can also be used to exceed activity cliffs [32]; moreover, side-effect based and off-label usage based similarities can be constructed for compounds using FDA-approved drugs as canonical bases in a group-representation [33].

Target-target similarities are another diverse and voluminous source of prior information, which can be defined using sequence similarities, common motifs and domains, phylogenetic relations or shared binding sites and pockets [71]. In case of indirect drug-target interactions, a broader set of target-target similarities could be based on relatedness in pathways, protein-protein networks and functional annotations, e.g. from Gene Ontology [76].

We concentrate on predicting presumably direct activities in this paper, thus we demonstrate the capability of the developed method and the effect multiple information sources using multiple chemical similarities, although the method can incorporate symmetrically multiple target-target similarities. Furthermore, the method can also incorporate separate prior expectations about the success rates of drugs in a given DTI, which could be combined with drug-likeliness [77], promiscuity prediction [78] and decoy prediction in case of their use [79]. Symmetrically, it can also incorporate separate prior expectations about the success rates of targets in a given DTI, which could be combined with druggability predictions [70, 80, 81] and the presence of pockets [82]. For an overview of available resources relevant for the DTI task, see e.g. [83, 84].

#### DTI methods

The rapid growth, especially the public availability of tabular (dyadic) DTI data in the last decade caused a dramatic shift of the applied statistical methods. For an overview of classical single prediction oriented machine learning and data mining in drug discovery, especially in DTI and ADME predictions, see e.g. [85], for large-scale, comprehensive applications of DTI data, see e.g. [86]. The tabular nature of the DTI data called for new methods not only handling this type of data natively, but also capable of using side information. Transfer learning and multitask learning paradigms addressed this challenge [8, 87, 88], but in the DTI context, two groups of methods, the pairwise conditional methods and the matrix factorization based generative methods proved to be particularly successful.

Pairwise conditional approaches or pairwise kernel methods flatten the dyadic structure of the DTI data and use drug and target descriptors, optionally even explanatory descriptors about the drug-target relations to predict interaction properties of drug-target pairs (for the assumptions behind the conditional approach, see e.g. [89], for its early DTI application, see e.g. [90]). Classification and regression methods, such as MLPs, decision trees and SVMs remain directly applicable in this conditional approach (not modeling the distribution of the drug-target pairs), however, the high number of drug-target pairs is challenging for kernel based methods [51, 91], but recent developments in deep learning show promising results [92]. Using multiple representations for drugs and targets is directly possible in this pairwise approach, but the construction of an aggregate pair-pair (interaction-interaction) similarity or an efficient set of pair-pair similarities from drug-drug and target-target similarities is an open problem. In the case of single drug-drug and target-target similarities, the Kroneckerian combination was proposed in the work of van Laarhooven [91] with corresponding computational simplifications to maintain scalability. Additionally, kernel techniques were extended to use multiple kernels, which are potentially derived from heterogeneous representations and similarities [51]. Recent extensions include non-linear kernel fusion in the RLS-KF system [50] and using boosting to learn from unscreened controls [54].

Matrix factorization (MF) methods differ from pairwise approaches in multiple properties crucial in the DTI task. The central operation of these methods is the construction of a joint space with latent factors for drugs and targets and modeling their interactions based on the inner product of the respective vectorial representations. Contrary, pairwise approaches, such as kernel methods or deep learning cannot directly exploit the tabular prior constraint of the data. The MF approach also allows the direct incorporation of drug-drug similarities and target-target similarities. Additionally, the low dimensionality of the latent space supports data visualization, although its interpretation is still in its infancy. Finally, probabilistic MF methods construct a distribution over the latent representations of drugs and targets, which in fact means that they are full-fledged generative models.

Matrix factorization methods were adopted early in gene expression data analysis [93, 94]. They were used for dimensionality reduction and the construction of a unified space for ligands and receptors [95], applied in biomedical text-mining and [96] and chemogenomics [97]. Later in the 2000’s media and e-commerce recommendation applications dominated the research of matrix factorization methods [98] and many developments were motivated and reported in these contexts, such as solutions for new items without interactions, selection bias, model regularization, automated parameter selection and incorporation of side information from multiple sources. An early work from Srebro et al. addressed the problems of using weights to represent importance or trust in the observations and the use of logistic regression as a non-linear transformation to predict probabilities of binary observations [99]. A special weighting of observations compared to unknowns were investigated in [100]. Salakhutdinov introduced Bayesian matrix factorization, which addressed regularization and automated parameter selection by Bayesian model averaging, also indicating the principled and flexible options for prior incorporation [101]. Severinski demonstrated the advantages of the full Bayesian approach versus a Maximum a Posteriori based alternative in this context [102]. Zhou introduced Gaussian process priors over the latent dimensions to enforce two kernels over row and column items [103]. Lobato et al. reported a variational Bayesian approach for logistic matrix factorization [104].

In the DTI context, an early kernel regression-based method (KRM) was reported in [7], and emphasized the advantages of a unified “pharmacological space”. Gönen introduced a kernelized Bayesian matrix factorization (KBMF) [22], which applies kernel-based averaging over the latent vectorial representations of rows and columns. The paper also introduced an efficient variational Bayesian approximation and indicated the interpretability of the latent space. Zheng et al. proposed a non-probabilistic multiple kernel learning approach, which achieved superior performance [23]. Multiple kernel learning was also realized in KBMF [27] and was also extended towards regression [105]. Special non-missing-at-random DTI data models were proposed in [52], which applied Gaussian priors to incorporate multiple kernels and used Gibbs sampling to approximate the posteriors. In an integrative work, Liu et al. proposed the combination of special neighborhood restricted kernels, network-based regularization, importance weights for the observations and logistic link functions in a non-Bayesian framework [48]. A recent extension applied a nonlinear kernel diffusion technique to boost relevant, complementary information in similarity matrices [49].

#### DTI benchmarks

The most widely used DTI benchmark from Yamanishi et al. [7] defined DTI prediction as a binary prediction problem with a single source of drug-drug and a target-target similarity, which induced the development of variety of methods and datasets (see Additional file 1). These datasets are still in the range of 1000×1000 and contain 10*k* interactions, but they inherit the problem of the selection bias present in the DTI repositories [11, 12, 65, 83, 106, 107]. Pahikkala et al. stressed the importance of fully observed bioactivity values in benchmarks [8], such as from Davis [9], to avoid misleading results because of selection bias, indirect interactions and the binary nature of the interactions. Liu et al. [48] reported a comprehensive evaluation of methods and released a corresponding benchmark implementation, the *pyDTI* package. For real, experimental evaluation of DTI methods, see e.g. [108, 109].

## Methods

Our work directly builds upon Gönen’s work on kernel-based matrix factorization using twin kernels (KBMF-MKL), which applied variational Bayesian approximations [27]. Another direct predecessor of our work is Liu et al’s neighborhood regularized logistic matrix factorization [48].

### Materials

To maintain consistency with earlier works, we evaluated the methods on the data sets provided by Yamanishi et al. [7] and Pahikkala et al. [8]. While the latter comes with multiple similarity matrices based on various molecular fingerprints, the former is one-kernel and therefore needed to be extended to properly test the MKL performance. We used the RDKit package [110] to compute additional MACCS and Morgan fingerprints for the molecules and used these in conjunction with the Tanimoto and Gaussian RBF similarity measures. Target similarities were obtained from Nascimento et al. [51] which utilized sequential, GO- and PPI-based similarities.

### Probabilistic model

Let **R**∈{0,1}^*I*×*J*^ denote the matrix of the interactions, where **R**_*ij*_=1 indicates a known interaction between the *i*th drug and *j*th target. In order to formulate a Bayesian model, we put a Bernoulli distribution on each **R**_*ij*_ with parameter $\sigma \left (\mathbf {u}_{i}^{T}\mathbf {v}_{j}\right)$ where *σ* is the logistic sigmoid function and **u**_*i*_, **v**_*j*_ are the *i*th and *j*th columns of the respective factor matrices $\mathbf {U} \in \mathbb {R}^{L\times I}$ and $\mathbf {V}\in \mathbb {R}^{L\times J}$. One can think of **u**_*i*_ and **v**_*j*_ as *L*-dimensional latent representations of the *i*th drug and *j*th target, and the *a posteriori* probability of an interaction between them is modeled by $\sigma \left (\mathbf {u}_{i}^{T}\mathbf {v}_{j}\right)$.

Similarly to NRLMF, we utilize an augmented version of the Bernoulli distribution parameterized by *c*≥1 which assigns higher importance to observations (positive examples). NRLMF also uses a post-training weighted average to infer interactions corresponding to empty rows and columns in **R** (i.e. these would have to be estimated without using any corresponding observations). We account for them by introducing variables **m** ^*u*^,**m** ^*v*^∈{0,1} indicating whether the row or column is empty. In these cases, only the side information will be used in the prediction. The conditional on the interactions can be written as

1 $$\begin{array}{*{20}l} {}p(\mathbf{R} \mid \mathbf{U}, \mathbf{V}, c, \mathbf{m}^{u}, \mathbf{m}^{v}) &\propto \prod_{i} \prod_{j} \left[ \left(\sigma\left(\mathbf{u}_{i}^{T}\mathbf{v}_{j}\right)\right)^{c\mathbf{R}_{ij}}\right.\\ & \quad\left.\left(1-\sigma\left(\mathbf{u}_{i}^{T}\mathbf{v}_{j}\right)\right)^{1-\mathbf{R}_{ij}} \right]^{\mathbf{m}^{u}_{i} \mathbf{m}^{v}_{j}}. \end{array} $$

Specifying priors on **U** and **V** presents an opportunity to incorporate multiple sources of side information. In particular, we can use a Gaussian distribution with a weighted linear combination of kernel matrices **K**_*n*_, *n*=1,2,… in the precision matrix, which corresponds to a combined *L*_2_-Laplacian regularization scheme [36]

2 $$\begin{array}{*{20}l} p(\mathbf{U} \!\mid\! \alpha^{u}\!,\! \mathbf{\gamma}^{u}\!,\! \mathbf{K}^{u})\! &\propto\! \prod_{i} \!\prod_{k} \!\exp\left\lbrace \!-\frac{1}{2}\sum_{n} \mathbf{\gamma}^{u}_{n} \mathbf{K}^{u}_{n,ik} \left\|\mathbf{u}_{i} - \mathbf{u}_{k} \right\|^{2} \right\rbrace \\& \quad\cdot\prod_{i} \exp\left\lbrace -\frac{\alpha^{u}}{2} \left\|\mathbf{u}_{i}\right\|^{2} \right\rbrace. \end{array} $$

The prior on **V** can be written similarly. To automate the learning of the optimal value of kernel weights $\mathbf {\gamma }^{u}_{n}$, we introduce another level of uncertainty using Gamma priors:

3 $$\begin{array}{*{20}l} p(\mathbf{\gamma}^{u}_{n} \mid a,b) = \frac{b^{a} (\mathbf{\gamma}^{u}_{n})^{a-1}e^{-b\mathbf{\gamma}^{u}_{n}}}{\Gamma(a)}. \end{array} $$

### Variational approximation

In the Bayesian approach, the combination of the data **R** and prior knowledge through kernel matrices **K**_*n*_ and hyperparameters defines the posterior

$$\begin{array}{*{20}l} p(\mathbf{U},\mathbf{V},\mathbf{\gamma}^{u},\mathbf{\gamma}^{v}|\mathbf{R},\mathbf{K}^{u}_{n},a^{u},b^{u},\mathbf{K}^{v}_{n},a^{v},b^{v},\alpha^{u},\alpha^{v},c). \end{array} $$

In the variational setting [111], we approximate the posterior with a variational distribution *q*(**U**,**V**,**γ** ^*u*^,**γ** ^*v*^). Suppressing the hyperparameters for notational simplicity, the expectation

$$\begin{array}{*{20}l} {}p(\mathbf{R})\!\! =\!\!\! \int\!\! p(\mathbf{R}\! \mid\! \mathbf{U},\! \mathbf{V}) p(\mathbf{U}\!\!\mid\!\mathbf{\gamma}\!^{u}) p(\mathbf{V}\!\mid\!\mathbf{\gamma}\!^{v}) p(\mathbf{\gamma}\!^{u}\!) p(\mathbf{\gamma}\!^{v}) d\mathbf{U} d\mathbf{V} d\mathbf{\gamma}\!^{u}\! d\mathbf{\gamma}^{v}\!, \end{array} $$

can be decomposed as

$$\begin{array}{*{20}l} \ln p(\mathbf{R}) = \mathcal{L}(q) + KL\left(q\mid\mid p\right), \end{array} $$

and, since the left hand side is constant with respect to *q*, maximizing the evidence lower bound $\mathcal {L}(q)$ with respect to *q* is equivalent to minimizing the Kullback–Leibler divergence *K* *L*(*q*∣∣*p*) between the variational distribution and the true posterior. In the mean field variational approach, maximization of $\mathcal {L}(q)$ is achieved by using a factorized variational distribution

$$\begin{array}{*{20}l} q\left(\mathbf{U},\mathbf{V},\mathbf{\gamma}^{u},\mathbf{\gamma}^{v}\right) = q(\mathbf{U})q(\mathbf{V})q\left(\mathbf{\gamma}^{u}\right)q\left(\mathbf{\gamma}^{v}\right). \end{array} $$

In particular, the evidence lower bound takes the form [112]

$${\begin{aligned} \mathcal{L}(q)\! \,=\,\! \int\!\! q(\mathbf{U})q(\mathbf{V})q(\mathbf{\gamma}^{u})q(\mathbf{\gamma}^{v}) \ln\! \!\left\lbrace\! \frac{p\left(\mathbf{R},\mathbf{U},\mathbf{V},\mathbf{\gamma}^{u},\mathbf{\gamma}^{v}\right)}{q(\mathbf{U})q(\mathbf{V})q\left(\mathbf{\gamma}^{u}\right)q\left(\mathbf{\gamma}^{v}\right)}\right\rbrace\! d\mathbf{U}d\mathbf{V}d\mathbf{\gamma}\!^{u} d\mathbf{\gamma}\!^{v}. \end{aligned}} $$

The optimal distribution *q* ^∗^(**U**) satisfies

$${\begin{aligned} \ln q^{*} (\mathbf{U}) &\,=\, E_{\mathbf{V},\mathbf{\gamma}^{u},\mathbf{\gamma}^{v}}\!\left[ \ln \!\left\lbrace p(\mathbf{R}\mid\mathbf{U},\mathbf{V}) p\!\left(\mathbf{U}\mid \mathbf{\gamma}^{u}\right) p\!\left(\mathbf{V}\mid \mathbf{\gamma}^{v}\right) \!p\left(\mathbf{\gamma}^{u}\right)\! p\left(\mathbf{\gamma}^{v}\right)\! \right\rbrace \right]\\ & \quad + \mathrm{const.} \end{aligned}} $$

which is non-conjugate due to the form of *p*(**R**∣**U**,**V**) and therefore the integral is intractable. However, by using Taylor approximation on the symmetrized logistic function (Jaakkola’s bound [104, 113])

$$\begin{array}{*{20}l} {}\sigma\!(z) \!\ge\! \tilde\sigma\!(z,\!\xi)\! =\! \sigma\!(\xi) \exp\! \left\lbrace\! \frac{z-\xi}{2}\! -\! \frac{1}{2\xi}\left(\!\sigma\!(\xi)\,-\,\frac{1}{2}\!\right) \!\left(z^{2} - \xi^{2} \right) \right\rbrace, \end{array} $$

we can lower bound *p*(**R**∣**U**,**V**) at the cost of introducing local variational parameters **ξ**_*ij*_, yielding a new bound $\mathcal {\tilde L}$ which contains at most quadratic terms. Collecting the terms containing **U** gives (see the proof in Additional file 2):

$$\begin{array}{*{20}l} {}\ln q^{*}(\mathbf{U}) \,=\, &-\!\frac{1}{2}\! \,\text{tr}\!\, \left(\!\mathbf{U}^{T}\! \mathbf{Q}^{u} \mathbf{U}\right)\! +\! \sum_{i} \mathbf{u}_{i}^{T}\! \left(\!\sum_{j} \hat{\mathbf{R}}_{ij} \hat{\mathbf{\xi}}_{ij} E\left[ \mathbf{v}_{j} \mathbf{v}_{j}^{T}\right] \right)\! \mathbf{u}_{i} \\&+ \sum_{i} \mathbf{u}_{i}^{T} \left(\sum_{j} \mathbf{R'}_{ij} E\left[\mathbf{v}_{j}\right] \right) \end{array} $$

where

$$\begin{array}{*{20}l} \mathbf{Q}^{u} &= \frac{E\left[\gamma_{u}\right]}{2} \left({\mathbf{K}^{u}}^{T}\mathbf{1} - \mathbf{K}^{u}\right) + \frac{\alpha_{u}}{2} \mathbf{I}, \\ \hat{\mathbf{\xi}}_{ij} &= -\frac{1}{2\mathbf{\xi}_{ij}} \left(\sigma(\mathbf{\xi}_{ij})-\frac{1}{2}\right),\\ \hat{\mathbf{R}}_{ij} &= \mathbf{m}^{u}_{i} \mathbf{m}^{v}_{j} \left((c - 1) \mathbf{R}_{ij} + 1 \right),\\ \mathbf{R'}_{ij} &= \mathbf{m}^{u}_{i} \mathbf{m}^{v}_{j} c \mathbf{R}_{ij} + \frac{1}{2} \hat{\mathbf{R}}_{ij}. \end{array} $$

Since this expression is quadratic in vec(**U**), we conclude that *q* ^∗^ is Gaussian and the parameters can be found by completing the square. In particular,

4 $$\begin{array}{*{20}l} {}q^{*}(\text{vec}(\mathbf{U})) &= \mathcal{N} (\text{vec}(\mathbf{U})\mid \mathbf{\phi}, \mathbf{\Lambda}^{-1}) \\ \mathbf{\Lambda} &= \mathbf{Q}^{u}\otimes\mathbf{I} - 2 \cdot \text{blkdg}_{i} \left(\sum_{j} \hat{\mathbf{R}}_{ij} \hat{\mathbf{\xi}}_{ij} E\left[ \mathbf{v}_{j} \mathbf{v}_{j}^{T}\right] \right), \end{array} $$

5 $$\begin{array}{*{20}l} \mathbf{\phi} &= {\boldsymbol{\Lambda}}^{-1} \text{vec}_{i} \left(\sum_{j} \mathbf{R'}_{ij} E\left[\mathbf{v}_{j}\right] \right), \end{array} $$

where blkdg_*i*_ denotes the operator creating an *L*·*I*×*L*·*I* block-diagonal matrix from *I* *L*×*L*-sized blocks. The variational update for *q*(**V**) can be derived similarly. The most computationally intensive operation is computing

6 $$\begin{array}{*{20}l}  E\left[ \mathbf{v}_{j} \mathbf{v}_{j}^{T}\right] = \text{Cov}(\mathbf{v}_{j}) + E\left[\mathbf{v}_{j}\right]E\left[\mathbf{v}_{j}\right]^{T} \end{array} $$

which requires the inversion of the precision matrix, performed using blocked Cholesky decomposition.

The optimal value of the local variational parameters **ξ**_*ij*_ can be computed by writing the expectation of the joint distribution in terms of **ξ** and setting its derivative to zero. In particular,

$$\begin{array}{*{20}l} \mathcal{\tilde L}(\mathbf{\xi}) &= \sum_{i} \sum_{j} \hat{\mathbf{R}}_{ij} \left(\ln \sigma(\mathbf{\xi}_{ij}) - \frac{\mathbf{\xi}_{ij}}{2} - \frac{1}{2\mathbf{\xi}_{ij}} \left(\sigma(\mathbf{\xi}_{ij}) - \frac{1}{2}\right)\right.\\&\quad\times\left. \left(\mathbf{\xi}_{ij}^{2} - E\left[\left(\mathbf{u}_{i}^{T}\mathbf{v}_{j}\right)^{2}\right]\right)\right), \end{array} $$

from which [104, 112]

7 $$ {\begin{aligned} \mathbf{\xi}_{ij}^{2} &= E\left[\left(\mathbf{u}_{i}^{T} \mathbf{v}_{j}\right)^{2}\right] \\ &= \left(E\left[ \mathbf{u}_{i}\right]^{T} E\left[ \mathbf{v}_{j}\right]\right)^{2} + \sum_{l} E\left[ \mathbf{U}_{li}\right]^{2} V\left[ \mathbf{V}_{lj} \right]+ V\left[ \mathbf{U}_{li} \right] E\left[ \mathbf{V}_{lj}\right]^{2} \\&\quad+ V\left[ \mathbf{U}_{li} \right] V\left[ \mathbf{V}_{lj} \right]. \end{aligned}}  $$

**Fig. 2:**
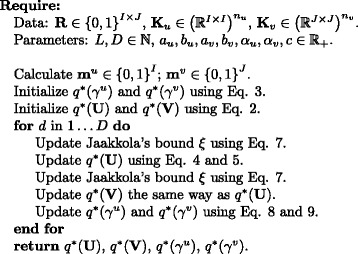
Pseudocode of the VB-MK-LMF algorithm

Since the model is conjugate with respect to the kernel weights, we can use the standard update formulas for the Gamma distribution

8 $$\begin{array}{*{20}l} q^{*}(\mathbf{\gamma}^{u}_{n}) &= \mathcal{G}amma(\mathbf{\gamma}^{u}_{n} \mid a',b') \\ a' &= a + \frac{I^{2}}{2} \end{array} $$

9 $$\begin{array}{*{20}l} b' &= b + \frac{1}{2} E_{\mathbf{U}} \left[ \sum_{i} \sum_{k} \mathbf{K}^{u}_{n,ik} \left\| \mathbf{u}_{i} - \mathbf{u}_{k}\right\|^{2} \right] \\ &= b + \frac{1}{2} \sum_{i} \sum_{k} \mathbf{K}^{u}_{n,ik} \left(E\left[\mathbf{u}_{i}^{T}\mathbf{u}_{i}\right] - 2E\left[\mathbf{u}_{i}^{T}\mathbf{u}_{k}\right]\right. \\ &\left.\quad + E\left[\mathbf{u}_{k}^{T}\mathbf{u}_{k}\right]\right), \end{array} $$

which also requires the explicit inversion of **Λ**. Figure 2 shows the pseudocode of the algorithm.

## Results

We present the results of a systematic comparison with KBMF-MKL [27], NRLMF [48] and KronRLS-MKL [51] using their provided implementations. Subsequently, our results show the effect of prior knowledge fading with increasing data size.

### Experimental settings

Predictive performance was evaluated in a 5 × 10-fold cross-validation framework. To maintain consistency with the evaluations in earlier works, we utilized the CVS1-CVS2-CVS3 settings as presented in [48] and calculated the average AUROC and AUPRC values in each scenario. In particular, CVS1 corresponds to evaluating predictive performance after randomly blinding 10*%* of the interactions and using them as test entities. CVS2 corresponds to random drugs (entire rows blinded) and CVS3 corresponds to random targets. We used the same folds as the PyDTI tool to maximize comparability.

In the single-kernel setting, we compared the performance of the proposed method to KBMF, NRLMF and KronRLS. The optimal parameters for NRLMF were obtained from the original publication [48]. KBMF and KronRLS were parameterized using a grid search method. VB-MK-LMF was used with 3 neighbors in each kernel, *α*_*u*_=*α*_*v*_=0.1, *a*_*u*_=*a*_*v*_=1, *b*_*u*_=*b*_*v*_=10^3^ and *c*=10. The number of latent factors was set to *L*=10 in the Nuclear Receptor dataset and *L*=15 in the others, and a more detailed investigation of this parameter was also conducted. The number of iterations was chosen manually as 20 since the variational parameters usually converged between 20−50 iterations.

In the multiple-kernel setting, we compared the performance of the proposed method to KBMF-MKL and KronRLS-MKL using MACCS and Morgan fingerprints with RBF and Tanimoto similarities. Target kernels provided by KronRLS-MKL did not improve the results in either case, thus only the ones computed by Yamanishi et al. were utilized. We also investigated the weights assigned to the kernels and tested robustness by introducing kernels with random values.

### Systematic evaluation

Single-kernel results are shown in Table 1. In most cases, VB-MK-LMF significantly outperforms NRLMF and one-kernel KBMF in terms of AUROC and AUPRC according to a pairwise *t*-test. Overall, the improvement is more modest on the Enzyme dataset, although still significant in some cases. This can be attributed to the fact that this dataset is by far the largest, which can mitigate the benefits of Bayesian model averaging and side information. On average, VB-MK-LMF yields 4.7*%* higher AUPRC values in the pairwise cross-validation setting than the second best method. In the drug and target settings, this is 2*%* and 7.6*%*, respectively. The lower AUROC and AUPRC values in these scenarios are explained by the lack of observations for the test drugs or targets in the training set, resulting in a harder task than in the pairwise scenario.

**Table 1:** Single-kernel results on gold standard data sets (maximum values are denoted by bold face)

	VB-MK-LMF	NRLMF	KBMF
AUROC (CV1)			
Nuclear Receptor	**0** **.** **9** **5** **7**±0.010	0.949±0.011	0.860±0.024
GPCR	**0** **.** **9** **7** **6**±0.003	0.960±0.004	0.911±0.004
Ion Channel	**0** **.** **9** **8** **9**±0.001	0.984±0.002	0.941±0.003
Enzyme	**0** **.** **9** **8** **7**±0.001	0.976±0.002	0.887±0.003
Kinase	**0** **.** **9** **2** **1**±0.002	0.919±0.001	0.916±0.001
AUPRC (CV1)			
Nuclear Receptor	**0** **.** **7** **7** **3**±0.030	0.723±0.042	0.533±0.047
GPCR	**0** **.** **7** **7** **7**±0.016	0.703±0.023	0.541±0.012
Ion Channel	**0** **.** **9** **1** **6**±0.007	0.863±0.012	0.763±0.009
Enzyme	**0** **.** **8** **9** **0**±0.006	0.876±0.007	0.656±0.008
Kinase	**0** **.** **8** **5** **0**±0.003	0.845±0.003	0.844±0.003
AUROC (CV2)			
Nuclear Receptor	**0** **.** **9** **3** **9**±0.021	0.896±0.023	0.845±0.023
GPCR	0.878±0.014	**0** **.** **8** **8** **3**±0.012	0.847±0.018
Ion Channel	**0** **.** **8** **1** **2**±0.026	0.800±0.026	0.785±0.021
Enzyme	**0** **.** **8** **5** **1**±0.021	0.811±0.024	0.718±0.028
Kinase	**0** **.** **8** **9** **4**±0.004	0.891±0.004	0.838±0.004
AUPRC (CV2)			
Nuclear Receptor	**0** **.** **5** **9** **3**±0.058	0.547±0.053	0.447±0.048
GPCR	**0** **.** **3** **6** **8**±0.023	0.363±0.023	0.365±0.024
Ion Channel	**0** **.** **3** **4** **5**±0.035	0.343±0.033	0.287±0.035
Enzyme	0.349±0.042	**0** **.** **3** **6** **0**±0.041	0.269±0.037
Kinase	**0** **.** **8** **0** **3**±0.009	0.797±0.010	0.735±0.009
AUROC (CV3)			
Nuclear Receptor	**0** **.** **9** **1** **7**±0.026	0.847±0.029	0.735±0.050
GPCR	**0** **.** **9** **4** **1**±0.009	0.920±0.014	0.839±0.020
Ion Channel	**0** **.** **9** **6** **6**±0.007	0.958±0.008	0.911±0.012
Enzyme	**0** **.** **9** **6** **2**±0.005	0.947±0.006	0.859±0.012
Kinase	**0** **.** **7** **6** **7**±0.018	0.763±0.018	0.740±0.022
AUPRC (CV3)			
Nuclear Receptor	**0** **.** **6** **0** **1**±0.081	0.456±0.079	0.352±0.070
GPCR	**0** **.** **5** **9** **6**±0.040	0.553±0.040	0.437±0.047
Ion Channel	**0** **.** **8** **2** **6**±0.021	0.788±0.028	0.695±0.024
Enzyme	0.794±0.017	**0** **.** **8** **0** **8**±0.018	0.573±0.028
Kinase	**0** **.** **6** **0** **8**±0.039	0.597±0.038	0.594±0.039

CV indicates the cross-validation setting (pairwise, drug and target, respectively). AUROC and AUPRC values were averaged over 5×10 runs and 95*%* confidence intervals were computed. In most cases, VB-MK-LMF significantly outperforms the other methods using *t*-test

**Fig. 3:**
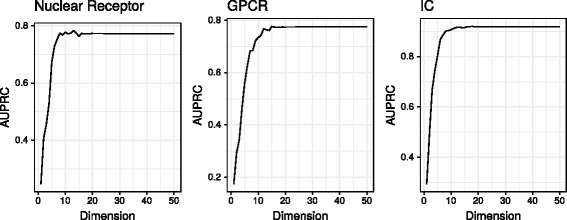
AUPRC values on the three smallest datasets with varying number of latent factors. The results become saturated around 10 dimensions

Following earlier investigations, we examined the number of latent factors, which has a crucial role from computational, statistical and interpretational aspects. Contrary to earlier works [44], which recommend 50−100 as the number of latent factors, we found that these values do not yield better results; in fact, the AUPRC values quickly become saturated. Conceptually, it is unclear what is to be gained going beyond the rank of the original matrix, which corresponds to perfect factorization with respect to the Frobenius norm when using SVD, and is also known to lead to serious overfitting in unregularized cases [99, 101]. Although overfitting is usually less of an issue with variational Bayesian approximations, a large number of latent factors significantly increases computational time. Figure 3 depicts the AUPRC values on the smaller datasets with varying number of latent factors. The Enzyme and Kinase datasets were not included in this experiment due to the rapidly increasing runtime.

Multi-kernel AUPRC values are shown in Table 2. Compared to the previous Table, it is clear that both VB-MK-LMF and KBMF benefits from using multiple kernels. Moreover, there is also an improvement in predictive performance when one combines instances of the same kernel but with different neighbor truncation values. However, advantages of using both of these combination schemes simultaneously are unclear as the results usually do not improve or even get worse (except for the Kinase dataset). This is a known property of linear kernel combinations, i.e. using large linear kernel combinations may not improve predictive performance beyond that of the best individual kernels in the combination [114].

**Table 2:** Multiple Kernel AUPRC values on gold standard data sets in the pairwise cross-validation setting (maximum values are denoted by bold face (maximum values are denoted by bold face)

Neighbors	MrgRbf	MrgTan	McsRbf	McsTan	Orig	All
Nuclear Receptor (KBMF-MKL: 0.566, KronRLS-MKL: 0.522)						
2	0.749	0.758	0.742	0.735	0.754	**0** **.** **7** **7** **9**
3	0.744	0.771	0.761	0.734	0.773	0.775
5	0.732	0.757	0.739	0.724	0.755	0.756
2+3	0.750	0.765	0.754	0.736	0.757	0.758
2+3+5	0.760	0.765	0.740	0.738	0.764	0.760
GPCR (KBMF-MKL: 0.622, KronRLS-MKL: 0.696)						
2	0.743	0.759	0.754	0.762	0.764	0.793
3	0.755	0.774	0.772	0.780	0.777	**0** **.** **8** **0** **2**
5	0.762	0.787	0.782	0.783	0.787	0.796
2+3	0.763	0.782	0.781	0.786	0.785	**0** **.** **8** **0** **2**
2+3+5	0.777	0.798	0.793	0.789	0.796	0.800
Ion Channel (KBMF-MKL: 0.826, KronRLS-MKL: 0.885)						
2	0.909	0.911	0.910	0.911	0.910	0.909
3	0.911	0.914	0.915	0.914	0.912	0.916
5	0.915	0.914	0.913	0.916	0.916	**0** **.** **9** **1** **7**
2+3	0.912	0.914	0.916	0.914	0.913	0.909
2+3+5	0.912	0.915	0.915	0.915	0.916	0.906
Enzyme (KBMF-MKL: 0.704, KronRLS-MKL: 0.893)						
2	0.885	0.887	0.879	0.883	0.888	0.884
3	0.885	0.890	0.885	0.882	0.890	**0** **.** **8** **9** **5**
5	0.883	0.886	0.880	0.881	0.884	0.883
2+3	0.888	0.889	0.880	0.881	0.888	0.881
2+3+5	0.887	0.889	0.881	0.878	0.888	0.875
Kinase (KBMF-MKL: 0.846, KronRLS-MKL: 0.561)						
Neighbors	-		2D	3D	ECFP	All
2			0.850	0.849	0.849	0.850
3			0.850	0.848	0.850	0.851
5	-		0.850	0.849	0.850	0.851
2+3			0.850	0.850	0.850	0.853
2+3+5			0.851	0.851	0.850	**0** **.** **8** **5** **4**

The table headers indicate the best AUPRC values obtained using the KBMF-MKL and KronRLS-MKL tools, utilizing all kernels and a grid search method for parameterization. The table bodies show AUPRC values from the VB-MK-LMF method in a cumulative manner. In particular, rows correspond to the cut-off value of the number of closest neighbors and the combinations of the resulting truncated kernels. Columns correspond to individual kernels. The last column was obtained by combining all kernels

Table 3 shows the normalized kernel weights in each of the datasets. For illustration purposes, we also included a unit-diagonal positive definite kernel matrix with random values. In the first four datasets, the algorithm assigned more or less uniform weights to the real kernels and a lower one to the random kernel. In the Kinase dataset, the random kernel is almost zeroed out. This underlines the validity of VB-MK-LMF’s kernel combination scheme. Setting *L* to *I* (the rank of the kernels) yields an almost zero weight to the random kernel, i.e. allowing larger dimensions also allows sufficient separation of the latent representations, which makes spotting kernels with erroneous values easier for the algorithm. This property might also justify increasing the number of latent factors beyond the rank of the interaction matrix in the multi-kernel setting.

**Table 3:** Normalized kernel weights with an extra positive definite, unit-diagonal, random valued kernel matrix

	MrgRbf	MrgTan	McsRbf	McsTan	Orig	Random
Nuclear Receptor	0.175	0.176	0.175	0.175	0.175	0.123
GPCR	0.173	0.173	0.172	0.172	0.172	0.138
Ion Channel	0.176	0.176	0.176	0.176	0.176	0.120
Enzyme	0.176	0.176	0.176	0.176	0.176	0.119
	-		2D	3D	ECFP	Random
Kinase	-		0.300	0.283	0.398	0.019

The number of latent factors was not altered in this experiment. Setting the number of latent factors to *I* (the rank of the kernel matrix) zeroes out the weight of the random kernel

**Fig. 4:**
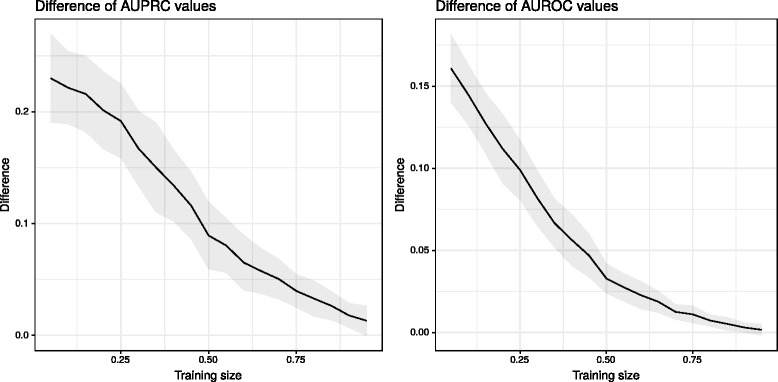
The effect of priors on predictive performance with varying sample sizes. The difference between the values using and not using kernels gradually vanishes as the training size increases. 95*%* confidence intervals are indicated by gray ribbons

To understand the effect of priors behind the significantly improved performance, which is especially pronounced at smaller sample sizes, we investigated the difference in AUPRC and AUROC values while using and ignoring kernels, at varying training set sizes. The results suggest the existence of a “small sample size” region where using side information offer significant gains, and after which the effect of priors gradually vanishes. Figure 4 depicts the learning curves.

## Discussion

VB-MK-LMF introduces a matrix factorization model incorporating multiple kernel learning, Laplacian regularization and the explicit modeling of interaction probabilities, for which a variational Bayesian inference method is proposed. The algorithm maps each drug and target into a joint vector space and interaction probabilities are derived from the inner products of the latent representations. Despite the suggested applicability of the unified “pharmacological space” [7], its semantics is still unexplored (for an early application in a ligand-receptor space, see [95], for a proof-of-concept illustration, see [22]). To facilitate a deeper understanding, we provide visual analytics tools alongside the factorization algorithm and allow arbitrary annotations to be mapped onto the latent representations.

**Fig. 5:**
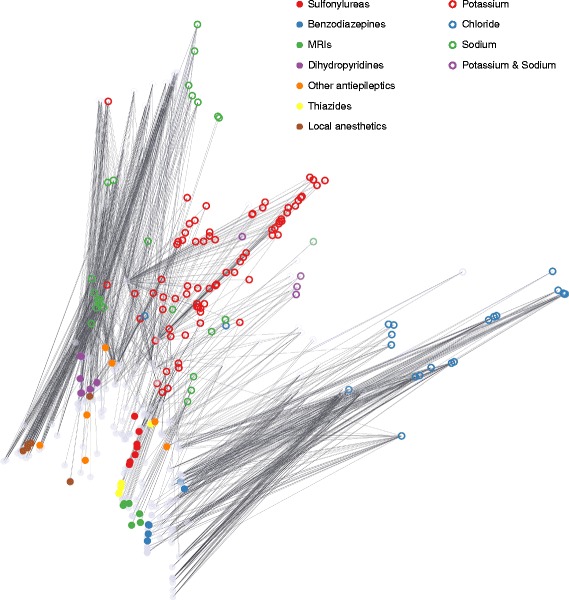
Latent representations of drugs and targets in the Ion Channel dataset using 2 latent dimensions. Drugs are colored on the basis of their respective ATC classes and targets are colored according to their ion transporter activity as obtained from the Gene Ontology. Known interactions are represented as edges

We demonstrate this on the Ion Channel dataset. Using *L*=2, the resulting latent representations can be visualized in a 2D Cartesian coordinate system as shown in Fig. 5. Drugs are colored on the basis of their respective ATC classes, where only the classes with more than 5 members were used. Targets are colored according to their ion transporter activity as obtained from the Gene Ontology. Known interactions are represented as edges. Even in this low-dimensional case, drugs in the same class tend to cluster together. The only exception is the “Other antiepileptics” class, which is easily explained by its heterogeneity, also indicated by the name. Targets also cluster fairly nicely, albeit with somewhat more outliers. It can be also observed that the targets exhibiting potassium and sodium transporter activity are placed halfway between the sodium and potassium groups.

**Fig. 6:**
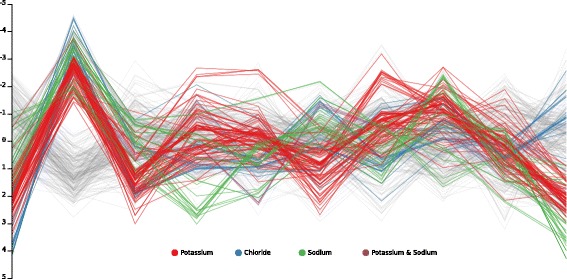
Parallel coordinates visualization of 10 latent dimensions in the Ion Channel dataset. Each curve corresponds to a latent representation of a drug or a target. Targets are colored on the basis of their ion transporter activity

Similarly, Fig. 6 depicts the joint space using a parallel coordinates visualization with *L*=10, where ion transporter activity is denoted by different colors. Most of the dimensions tend to separate at least one class from the others and many of them seem to distinguish between more than two classes. This indicates that the algorithm manages to find biologically meaningful latent dimensions, possibly encoding pharmacophore properties and the properties of binding sites, but we leave it for further exploration.

**Fig. 7:**
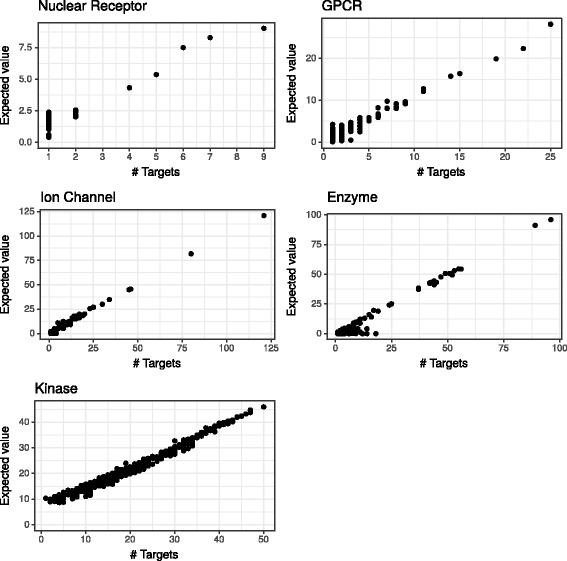
Drug promiscuity vs. the expected number of interactions. The number of targets of each drug in the datasets are depicted on the horizontal axis. The expected number of interactions as predicted by VB-MK-LMF are depicted on the vertical axis

**Fig. 8:**
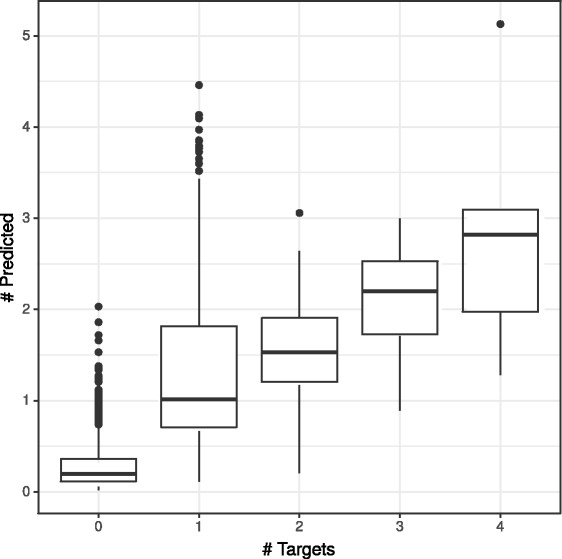
Expected number of interactions as predicted by VB-MK-LMF for each drug in the GPCR dataset. The number of targets are depicted on the horizontal axis. A 10× cross-validation setting was used

From a more practical viewpoint, it is important to touch on the issue of drug promiscuity and polypharmacology. This refers to the observation that some drugs tend to act on multiple targets leading to distinct pharmacological effects, which is often considered an undesirable property [86], although partly unavoidable and potentially utilizable [115]. In either case, predicting the expected number of interactions in a restricted set of targets is a unique property of probabilistic DTI predictors, e.g. compared to ranking approaches. To illustrate this ability of VB-MK-LMF, we computed the expected value of the total number of interactions for every drug in all datasets, treating them independently, shown in Fig. 7 together with the number known targets. Overall, the expected value of further hits approximates the number of interactions already discovered rather closely, although it tends to over-estimate, especially when only one or two interactions are known. We also conducted a 10 × cross-validation experiment for each drug in the GPCR dataset and performed the same comparison with similar results (Fig. 8). It is worth to mention that the number of currently unobserved positive interactions in large-scale settings and in comprehensive DTI repositories is vital for the pharmaceutical industry and an open scientific question, as indicated by research on drug-likeliness and druggability. Assuming total independence, the expected value provides a raw estimate for this. However, as the relative frequency of positive interactions among the unobserved cases should influence the selection of weight for the observed cases (*c*), and the value of *c* influences the expected value, resolving this circular situation and tuning *c* requires further investigations.

We also performed a case-based evaluation by obtaining the top 5 novel predictions in the incomplete datasets and examining whether they are present in the current version of the DrugBank database. Most interactions were confirmed and some of the unconfirmed hits are known to bind to other members of that particular protein family. This shows the ability of VB-MK-LMF to predict novel interactions. The predicted lists are similar to those of the NRLMF method. Table 4 illustrates these results and also contains the rank of the predicted interactions among the NRLMF predictions.

**Table 4:** Top 5 predicted interactions which are not present in the datasets

Probability	Drug	Target	Drug name	Target name	DrugBank	NRLMF
Nuclear Receptor						
0.943	D00316	hsa6096	Etretinate	RARB	Yes	1
0.671	D01132	hsa6097	Tazarotene	RARC	^a^RARB	6
0.662	D01132	hsa190	Tazarotene	NR0B1		18
0.529	D00898	hsa2100	Dienestrol	ESR2	Yes	7
0.445	D00094	hsa6095	Tretinoin	RARA	Yes	26
GPCR						
0.966	D00283	hsa1814	Clozapine	DRD3	Yes	1
0.956	D00110	hsa1813	Cocaine	DRD2		188
0.938	D02358	hsa154	Metoprolol	ADRB2	Yes	2
0.937	D02614	hsa154	Denopamine	ADRB2	Yes	4
0.937	D04625	hsa154	Isoetharine	ADRB2	Yes	3
Ion Channel						
0.990	D00538	hsa6331	Zonisamide	SCN5A	Yes	9
0.986	D00294	hsa3767	Diazoxide	KCNJ11	Yes	244
0.985	D00552	hsa6331	Tetracaine	SCN5A	Yes	5
0.983	D00438	hsa779	Nimodipine	CACNA1S	Yes	2
0.983	D00649	hsa8911	Amiloride	CACNA1I		83
Enzyme						
0.999	D00542	hsa1571	Halothane	CYP2E1	Yes	1
0.995	D00097	hsa5743	Salicylic acid	PTGS2	Yes	4
0.995	D00437	hsa1559	Nifedipine	CYP2C9	Yes	5
0.987	D00501	hsa50940	Pentoxifylline	PDE11A	^a^PDE5A	2
0.986	D00501	hsa5150	Pentoxifylline	PDE7A	^a^PDE5A	3

Many of the hits were confirmed by the current version of DrugBank. The ^a^symbol indicates a known interaction with another member of the protein family. The last column denotes the rank of the interaction among the NRLMF predictions

**Fig. 9:**
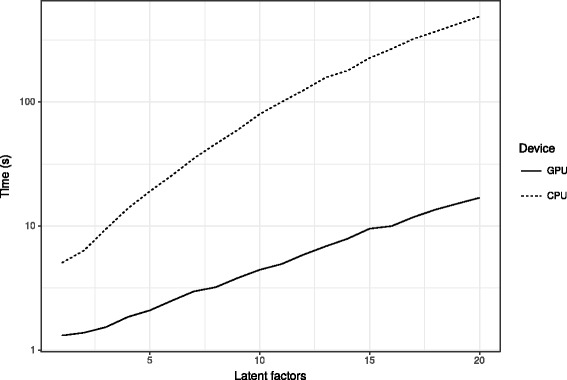
Runtime of the GPU and CPU implementations in terms of the number of latent factors. This benchmark was conducted on a 200×200 matrix factorization. The GPU implementation brings a 30× speedup on an NVIDIA GTX Titan X graphics card

Finally, we discuss computational issues. Due to the explicit computation of inverse matrices, the variational approximation is highly compute-intensive, however, it is straightforward to parallelize and many steps can be written as BLAS operations. GPUs are particularly well-suited for this task. All computations presented in this work can be performed on a mid-range graphics card. Figure 9 shows the runtime of GPU and CPU implementations in terms of latent factors 200×200 matrix factorization task, which showed a 30× speedup using an NVIDA Titan X graphics card. However, in larger dimensions or with many latent factors, one can quickly run out of GPU memory, i.e. scaling remains an open question. Although GPUs provide excellent performance with single precision, double precision performance typically lags far behind, especially with modern consumer-level graphics cards. This raises the issue of numerical stability. To cope with the memory footprint of the algorithm, we provide a sparse implementation beside the standard dense solver. To address the issue of numerical stability, we also provide a QR factorization-based implementation which is more stable but significantly slower than the default Cholesky-based method. The computation in VB-MK-LMF is dominated by the inversion in Eq. 6, which gives $\mathcal {O}(D L^{3}\max (I^{3},J^{3}))$ for the total time complexity (*D* is the number of iterations). Comparison with the time complexity of NRLMF, $\mathcal {O}(D L I J)$, clearly shows the burden of Bayesian computation in the current implementation and calls for the usage of approximative inversion techniques, which we consider as a future work.

## Conclusion

We presented Variational Bayesian Multiple Kernel Logistic Matrix Factorization (VB-MK-LMF), integrating multiple kernel learning, weighted observations, graph Laplacian regularization, and explicit modeling of probabilities of binary drug-target interactions. Compared to other state-of-the-art methods, VB-MK-LMF achieved significantly better predictive performance in standard benchmarks.

Admittedly, benchmarking the pure predictive performance on a given dataset gives a very focused view about the real-world applicability of the methods, but helps comparability. On the other hand, the release of new and updated datasets as shown in Additional file 1 in fact quickly create an impractical fragmentary situation. In general, the definition of a standard background knowledge pool for a benchmarking is even more complicated, as earlier attempts show in computational fusion methods for gene prioritization [116, 117].

Additionally, currently the possible utilizations of a DTI prediction method in real-world applications are at least as diverse as the methodological repertoire. For example, DTI prediction methods could be applied in data quality control phase for anomaly detection, especially in the case of merging different bioactivity values from public and private sources. Screening design, hit triage and prioritization for further validation [118], possibly in an active learning framework [16, 119], are standard usages. Finally, DTI prediction methods may also provide essential data to support visualization and visual data analytics, as we demonstrated in a new range of dimensionality (10−20), which proved to be sufficient with VB-MK-LMF.

Another key property of VB-MK-LMF is the explicit modeling of probabilities, which allows the prediction of interaction probabilities and their credibility. We demonstrated the use of probabilistic predictions by proposing DTI dataset specific versions of promiscuity and druggability, through the expected number of hits in a dataset for a drug or a target respectively. In general, the predicted posteriors for the interactions can be seen as a probabilistic “data-analytic” knowledge base, which allows new functionalities in post-processing, beyond enrichment methods available for ranking methods [33, 37]. To utilize the Bayesian predictions of VB-MK-LMF, we also plan to investigate their decision theoretic usage, when certainty for expected gains and losses of prioritization of interactions is expected, e.g. in functional validations.

Further interesting research directions are the regression version of VB-MK-LMF directly approximating the continuous activity data [8, 52] and the use of multiple instances of VB-MK-LMF for overlapping DTI matrices, which are linked to each other by weighted common observations. The latter could improve the scalability of the method using parallel implementations for mid-sized DTI tasks with 10^5^ drugs and 10^4^ targets, going beyond the current benchmarks.

## Additional files

Additional file 1 The properties of DTI methods related to the development or evaluation of VB-MK-LMF. (PDF 124 kb)

Additional file 2 Derivation of the lower bound using Jaakkola’s bound on the logistic sigmoid. (PDF 107 kb)

## Abbreviations

ADME: Absorption, distribution, metabolism, and excretion
AUPRC: Area under the precision-recall curve
AUROC: Area under the receiver operating characteristic curve
CPI: Compound-protein interaction
CVS: Cross-validation setting
DTI: Drug-target interaction
FDA: Food and drug administration
GPCR: G-protein coupled receptor
GP-GPU: General purpose computing on graphics processing unit
KBMF: Kernelized Bayesian matrix factorization
KRM: Kernel regression-based method
MACCS: Molecular access system
MF: Matrix factorization
MKL: Multiple Kernel learning
MLP: Multi-layer perceptron
NRLMF: Neighborhood regularized logistic matrix factorization
RLS-KF: Regularized least squares kernel fusion
SVD: Singular value decomposition
SVM: Support vector machine
VB-MK-LMF: Variational Bayesian multiple kernel logistic matrix factorization
