# Supplementary material for: VB-MK-LMF: fusion of drugs, targets and interactions using variational Bayesian multiple kernel logistic matrix factorization
Source: BMC Bioinformatics. 2017 Oct 4;18:440. doi: 10.1186/s12859-017-1845-z (PMC5628496; doi:10.1186/s12859-017-1845-z)
Supplement: Supplementary file 1 — The properties of DTI methods related to the development or evaluation of VB-MK-LMF. (PDF 124 kb) [file 12859_2017_1845_MOESM1_ESM.pdf]

Name	Data				Prior					Method					Output				Status		
	Type	Size	Availability	Novel	De-bias	Side inf.	Preproc.	Usage	Numeric	Models	Pairwise	Matrix fact.	Cost/Posterior	Scalability	Prediction	Indirect	Latent repr.	Fusion	Availability	Benchmark	Re-evaluated
KRM [1]	B	M	1	P	-	O	-		N	M		R	N	Q*	R	-	1		S	Y	
BLM [2]	B	M	1	-	-	O	-		N	L	S		N	P^4	R	-			S	Y	
NetLapRLS [3]	B	M	1	-	-	O	-	L	N	M		D	N	C	R	-			-	Y	
Bipartite RankSVM [4]	B	S		-	-	-				L	S		R	P^4	R	-			-	-	
GIP-KronRLS [5]	B	M	1	-	E	O	-	P	N	P	S		F	C	R	-			-	≈Y	
SITAR [6]	B	M	-	-	R	O	-		N	P	L		L	L	R		1	W	-	Y	
SLAP [7]	B	O	1	F	-	N	N	-	L	G			-	≈C	R	1		W	W	-	
KBMF2MKL [8]	B	M	1	-	-	M	-	K	N	F		K	F	C	P	-	1	WC	S	Y	1
Yu et al. [9]	B	O	1	P	R	M	-	C		P	S		L	P^4	R	-	-	-	-	-	
NBSI [10]	B	O	1	P	-	O	-		N	G				Q	R				1	-	
BLM-NII [11]	B	M		-	-	O	-	N	N	L			F	C	P	-	-	-	-	Y	
WNN-GIP [12]	B	M		-	-	O	-	N	N	P	K		F	C	P	-	-	-	-	≈Y	
MSCMF [13]	B	M		-	-	M	-	-	N	M		D	F	C	R	-	-	W	-	Y	
RBM [14]	N	M	1	P	-	O-		C		P	B		L	≈L	P	1	1	-	S	-	
Macau [15]	R	O	1	N	-	D	-	C	N	M		D	P	L	D	-	1	-	S	-	
Meta-path [16]	G	O	1	F	-	N	-		L	S			R	≈C	R	1		W	S	-	
DrugE-rank [17]	B	M	1	F	-	O			N	P			R		R	-	-		S	-	
NRLMF [18]	B	M		-	W	O	N	L	N	M		L	L	Q	P	-	-		S	Y	1
DNILMF [19]	B	M	1	F	-	M	D	K	N	M		L	L	-	P	-	1	C	S	Y	
RLS-KF [20]	B	M		-	-	O	-	K	N	P	K		F		R	-			-	Y	
KronRLS-MKL [21]	B	M		-	-	M	-	K	N	P	K		F		R	-		W	S	Y	1
HuTol [22]	R	M		-	M	M	-	G	N	M		K	P	L	D	-	1	WC	S	-	
SDTNBI [23]	G	M	-	F	-	N			L	G					R	-			S	-	
SELF-BLM [24]	B	M		-	B	O	-		N	L	K		F		R	-			S	Y	

The properties of DTI methods related to the development or evaluation of VB-MK-LMF. Columns are the following. The empty cell denotes that the classification is not applicable or a false value in the binary case. The '-' sign denotes that none of the values is applicable. The ≈ sign indicates approximate applicability of the category.*Data* Type: (B)inary, (N)ominal, (R)eal(s), (G)raphs. Size: (S)ingle(s), (M)id(10^3x10^3), (O)mic(all). Availability: 1 denotes public availabilityNovel: (P)ositive only, (N)egative observations are also included, (F)ull systematic. *Prior* De-bias: (R)epresentativity (using predefined positive/unscreened negative ratio in training samples), (E)ncoding of outcome, (W)eights on outcome, NMAR (M)odel, semi-(B)oosting. Side information: About (D)rugs, (O)ne drug - one target kernel, (M)ultiple drug or target kernels, about (I)nteractions, (N)etworks. Preprocessing: (N)eighbourhood restriction, kernel (D)iffusion. Usage: (C)oncatenation of descriptions, (N)eighbourhoodness, (P)airwise kernel by Kronecker product, (G)aussain process prior/(K)ernelized/(L)aplacian regularization over latent representations. Numeric: (L)ogical priors only or (N)umeric as well. *Approach* Models: (L)ocal models, (P)airwise, (M)atrix approximations, Matrix (F)actorization, (G)raph-based, (S)emantic. Pairwise: (B)oltzmann machine, (K)ernel bases, (S)VM, (D)ecision tree, (Random) forest, (N)aive Bayesian network, (L)ogistic regression. Matrix factorization: (D)irect approximation; low rank approximations: (R)egression-based, (K)ernelized, (L)ogistic. Cost/Posterior: (F)robenius/squared, (L)ikelihood, (R)ank-based, Bayesian over (D)ecisions, Bayesian over (P)redictions. Scalability: Using the following notations: number of (training) drugs: I (I'), the number of (training) targets: J (J'), dimension of latent space in matrix factorization methods: L. (L)inear: $\mathcal{O}(|R|)$, (Q)uadratic: $\mathcal{O}(LIJ)$, (C)ubic: $\mathcal{O}(L^3 \max(I^3, J^3))$, P^4:

polynomial with degree $\mathcal{O}((I'J')^2)$, P^6: $\mathcal{O}((I'J')^3)$. *Output* Prediction: (L)abel, (R)ank/Score, (P)robability, (D)ensity. Indirect(1): capability of discriminating indirect interactions. Latent representation(1): for drugs and targets. Fusion: (W)eights for sources, (C)ombined kernel. *Status* Availability: (S)ource is publicly available, (W)eb service. Benchmarking: (Y)amanishi et al [1], (D)avis et al. [25]. Re-evaluated(1): re-evaluation in our paper.

Author details

References

1. Yamanishi, Y., Araki, M., Gutteridge, A., Honda, W., Kanehisa, M.: Prediction of drug-target interaction networks from the integration of chemical and genomic spaces. Bioinformatics **24**(13), 232–240 (2008). doi:10.1093/bioinformatics/btn162
2. Bleakley, K., Yamanishi, Y.: Supervised prediction of drug-target interactions using bipartite local models. Bioinformatics **25**(18), 2397–2403 (2009). doi:10.1093/bioinformatics/btp433
3. Xia, Z., Wu, L.-Y., Zhou, X., Wong, S.T.C.: Semi-supervised drug-protein interaction prediction from heterogeneous biological spaces. BMC systems biology **4**(S6), 6 (2010). doi:10.1186/1752-0509-4-S2-S6
4. Agarwal, S., Dugar, D., Sengupta, S.: Ranking chemical structures for drug discovery: A new machine learning approach. Journal of Chemical Information and Modeling **50**(5), 716–731 (2010). doi:10.1021/ci9003865
5. van Laarhoven, T., Nabuurs, S.B., Marchiori, E.: Gaussian interaction profile kernels for predicting drug-target interaction. Bioinformatics **27**(21), 3036–3043 (2011). doi:10.1093/bioinformatics/btr500
6. Perlman, L., Gottlieb, A., Atias, N., Ruppin, E., Sharan, R.: Combining Drug and Gene Similarity Measures for Drug-Target Elucidation. Computational Biology **18**(2), 133–145 (2011). doi:10.1089/cmb.2010.0213
7. Chen, B., Ding, Y., Wild, D.J.: Improving integrative searching of systems chemical biology data using semantic annotation. Journal of cheminformatics **4**(1), 6 (2012). doi:10.1186/1758-2946-4-6
8. Gönen, M., Khan, S., Kaski, S.: Kernelized bayesian matrix factorization. In: International Conference on Machine Learning, pp. 864–872 (2013)
9. Yu, H., Chen, J., Xu, X., Li, Y., Zhao, H., Fang, Y., Li, X., Zhou, W., Wang, W., Wang, Y.: A systematic prediction of multiple drug-target interactions from chemical, genomic, and pharmacological data. PLoS ONE **7**(5) (2012). doi:10.1371/journal.pone.0037608
10. Cheng, F., Liu, C., Jiang, J., Lu, W., Li, W., Liu, G., Zhou, W., Huang, J., Tang, Y.: Prediction of drug-target interactions and drug repositioning via network-based inference. PLoS Computational Biology **8**(5) (2012). doi:10.1371/journal.pcbi.1002503
11. Mei, J.P., Kwoh, C.K., Yang, P., Li, X.L., Zheng, J.: Drug-target interaction prediction by learning from local information and neighbors. Bioinformatics **29**(2), 238–245 (2013). doi:10.1093/bioinformatics/bts670
12. van Laarhoven, T., Marchiori, E.: Predicting Drug-Target Interactions for New Drug Compounds Using a Weighted Nearest Neighbor Profile. PLoS ONE **8**(6), 1–6 (2013). doi:10.1371/journal.pone.0066952
13. Zheng, W., Thorne, N., McKew, J.C.: Phenotypic screens as a renewed approach for drug discovery. Drug Discovery Today **18**(21-22), 1067–1073 (2013). doi:10.1016/j.drudis.2013.07.001
14. Wang, Y., Zeng, J.: Predicting drug-target interactions using restricted Boltzmann machines. Bioinformatics **29**(13), 126–134 (2013). doi:10.1093/bioinformatics/btt234
15. Simm, J., Arany, A., Zakeri, P., Haber, T., Wegner, J.K., Chupakhin, V., Ceulemans, H., Moreau, Y.: Macau: Scalable Bayesian Multi-relational Factorization with Side Information using MCMC. ArXiv e-prints (2015). 1509.04610
16. Fu, G., Ding, Y., Seal, A., Chen, B., Sun, Y., Bolton, E.: Predicting drug target interactions using meta-path-based semantic network analysis. BMC bioinformatics **17**(1), 160 (2016)
17. Yuan, Q., Gao, J., Wu, D., Zhang, S., Mamitsuka, H., Zhu, S.: DrugE-Rank: Improving drug-target interaction prediction of new candidate drugs or targets by ensemble learning to rank. Bioinformatics **32**(12), 18–27 (2016). doi:10.1093/bioinformatics/btw244
18. Liu, Y., Wu, M., Miao, C., Zhao, P., Li, X.L.: Neighborhood Regularized Logistic Matrix Factorization for Drug-Target Interaction Prediction. PLoS Computational Biology **12**(2), 1–26 (2016). doi:10.1371/journal.pcbi.1004760
19. Hao, M., Bryant, S.H., Wang, Y., Iorio, F., Rittman, T., Ge, H., Menden, M., Saez-Rodriguez, J., Bartlett, J.B., Dredge, K., Dalgleish, A.G., Steinbach, G., Koehl, G.E., Schlitt, H.J., Geissler, E.K., Cappelli, C., Gu, S., Keiser, M.J., Wang, L., Haupt, V.J., Schroeder, M., Ma, D.L., Chan, D.S., Leung, C.H., Yamanishi, Y., Araki, M., Gutteridge, A., Honda, W., Kanehisa, M., Bleakley, K., Yamanishi, Y., van Laarhoven, T., Nabuurs, S.B., Marchiori, E., Mei, J.-P., Kwoh, C.-K., Yang, P., Li, X.-L., Zheng, J., Hao, M., Wang, Y., Bryant, S.H., Wang, B., Liu, Y., Wu, M., Miao, C., Zhao, P., Li, X.L., Kanehisa, M., Schomburg, I., Günther, S., Wishart, D.S., Kuang, Q., Smith, T.F., Waterman, M.S., Hattori, M., Okuno, Y., Goto, S., Kanehisa, M., Ma, H., King, I., Lyu, M.R., Duchi, J., Hazan, E., Singer, Y., Gonen, M., Kaski, S., Cao, Y., Charisi, A., Cheng, L.-C., Jiang, T., Girke, T., Guha, R., Sievers, F., Leslie, C., Eskin, E., Noble, W.S., Langham, J.J., Cleves, A.E., Spitzer, R., Kirshner, D., Jain, A.N., Collins, I., von Coburg, Y., Kottke, T., Weizel, L., Ligneau, X., Stark, H., Wishart, D., Alaimo, S., Sui, J.: Predicting drug-target interactions by dual-network integrated logistic matrix factorization. Scientific Reports **7**(January), 40376 (2017). doi:10.1038/srep40376
20. Hao, M., Wang, Y., Bryant, S.H.: Improved prediction of drug-target interactions using regularized least squares integrating with kernel fusion technique. Analytica Chimica Acta **909**, 41–50 (2016). doi:10.1016/j.aca.2016.01.014. 15334406
21. Nascimento, A.C.a., Prudêncio, R.B.C., Costa, I.G.: A multiple kernel learning algorithm for drug-target interaction prediction. BMC bioinformatics **17**(1), 46 (2016). doi:10.1186/s12859-016-0890-3

22. Bolgár, B., Antal, P.: Bayesian matrix factorization with non-random missing data using informative Gaussian process priors and soft evidences. In: Antonucci, A., Corani, G., Campos, C.P. (eds.) Proceedings of the Eighth International Conference on Probabilistic Graphical Models, pp. 25–36 (2016)
23. Wu, Z., Cheng, F., Li, J., Li, W., Liu, G., Tang, Y.: SDTNBI: an integrated network and chemoinformatics tool for systematic prediction of drug–target interactions and drug repositioning. Briefings in Bioinformatics (October 2015), 012 (2016). doi:10.1093/bib/bbw012
24. Keum, J., Nam, H.: Self-blm: Prediction of drug-target interactions via self-training svm. PloS one **12**(2), 0171839 (2017)
25. Davis, M.I., Hunt, J.P., Herrgard, S., Ciceri, P., Wodicka, L.M., Pallares, G., Hocker, M., Treiber, D.K., Zarrinkar, P.P.: Comprehensive analysis of kinase inhibitor selectivity. Nature Biotechnology **29**(11), 1046–1051 (2011). doi:10.1038/nbt.1990. 0402594v3
