# Supplementary material for: VB-MK-LMF: fusion of drugs, targets and interactions using variational Bayesian multiple kernel logistic matrix factorization
Source: BMC Bioinformatics. 2017 Oct 4;18:440. doi: 10.1186/s12859-017-1845-z (PMC5628496; doi:10.1186/s12859-017-1845-z)
Supplement: Supplementary file 2 — Derivation of the lower bound using Jaakkola’s bound on the logistic sigmoid. (PDF 107 kb) [file 12859_2017_1845_MOESM2_ESM.pdf]

Proof The lower bound in $\mathbf{U}$ can be derived by using Jaakkola's bound on the logistic sigmoid and keeping the terms quadratic and linear in $\mathbf{U}$:

$$\begin{aligned}
\ln q^*(\mathbf{U}) =& E_{\mathbf{V},\gamma^u,\gamma^v}\left[\ln\left\{p(\mathbf{R} \mid \mathbf{U},\mathbf{V})p(\mathbf{U} \mid \gamma^u)\right\}\right] \\
=& -\frac{E\left[\gamma_u\right]}{2}\sum_i\sum_k \mathbf{K}_{ik}\left\|\mathbf{u}_i-\mathbf{u}_k\right\|^2 - \frac{\alpha_u}{2}\sum_i \mathbf{u}_i^T\mathbf{u}_i \\
& + c\sum_i\sum_j \mathbf{m}_i^u\mathbf{m}_j^v\mathbf{R}_{ij}\mathbf{u}_i^T E\left[\mathbf{v}_j\right] \\
& + \sum_i\sum_j E_{\mathbf{V}}\left[\mathbf{m}_i^u\mathbf{m}_j^v\left((c-1)\mathbf{R}_{ij}+1\right)\ln\sigma\left(-\mathbf{u}_i^T\mathbf{v}_j\right)\right] + \text{const.} \\
\geq& -\frac{E\left[\gamma_u\right]}{2}\sum_i\sum_k \mathbf{K}_{ik}\left\|\mathbf{u}_i-\mathbf{u}_k\right\|^2 - \frac{\alpha_u}{2}\sum_i \mathbf{u}_i^T\mathbf{u}_i + c\sum_i\sum_j \mathbf{m}_i^u\mathbf{m}_j^v\mathbf{R}_{ij}\mathbf{u}_i^T E\left[\mathbf{v}_j\right] \\
& + \sum_i\sum_j \mathbf{m}_i^u\mathbf{m}_j^v\left((c-1)\mathbf{R}_{ij}+1\right)\left(-\frac{\mathbf{u}_i^T E\left[\mathbf{v}_j\right]}{2} - \frac{1}{2\xi_{ij}}\left(\sigma(\xi_{ij})-\frac{1}{2}\right)\mathbf{u}_i^T E\left[\mathbf{v}_j\mathbf{v}_j^T\right]\mathbf{u}_i\right) \\
=& -\frac{1}{2}\operatorname{tr}\left(\mathbf{U}^T\mathbf{Q}^u\mathbf{U}\right) + \sum_i \mathbf{u}_i^T\left(\sum_j \hat{\mathbf{R}}_{ij}\hat{\xi}_{ij}E\left[\mathbf{v}_j\mathbf{v}_j^T\right]\right)\mathbf{u}_i + \sum_i \mathbf{u}_i^T\left(\sum_j \mathbf{R'}_{ij}E\left[\mathbf{v}_j\right]\right),
\end{aligned}$$

where

$$\begin{aligned}
\mathbf{Q}^u &= \frac{E\left[\gamma_u\right]}{2}\left(\mathbf{K}^{u^T}\mathbf{1}-\mathbf{K}^u\right)+\frac{\alpha_u}{2}\mathbf{I}, \\
\hat{\xi}_{ij} &= -\frac{1}{2\xi_{ij}}\left(\sigma(\xi_{ij})-\frac{1}{2}\right), \\
\hat{\mathbf{R}}_{ij} &= \mathbf{m}_i^u\mathbf{m}_j^v\left((c-1)\mathbf{R}_{ij}+1\right), \\
\mathbf{R'}_{ij} &= \mathbf{m}_i^u\mathbf{m}_j^v c\mathbf{R}_{ij}+\frac{1}{2}\hat{\mathbf{R}}_{ij}.
\end{aligned}$$

$\square$
